# Management of Oroantral Fistulae and Communications: Our Recommendations for Routine Practice

**DOI:** 10.1155/2021/7592253

**Published:** 2021-12-14

**Authors:** Mahamadou Konate, Dounia Sarfi, Mounia El Bouhairi, Ihsane Benyahya

**Affiliations:** Department of Oral Surgery, Université Hassan II, Casablanca, Morocco BP: 9157

## Abstract

Oroantral communication (OAC) is one of the main complications of extracting antral or sinus teeth. OAC is a gap between the maxillary sinus and the oral cavity. When left untreated, it causes maxillary sinusitis and dramatically impairs the patient's quality of life. Numerous surgical treatment techniques have been described, from mucosal techniques to using bone substitutes or very conservative alternative means. Many cases of recurrence after treatment have been reported, and the choice of the method adapted to the clinical situation remains challenging. Therefore, it is necessary to establish a correct diagnosis and choose the surgical technique best adapted to the case. This work is aimed at reviewing several techniques for the treatment of OAC and at describing our recommendations for use in daily practice for each of them through four clinical cases.

## 1. Introduction

**Figure 1:**
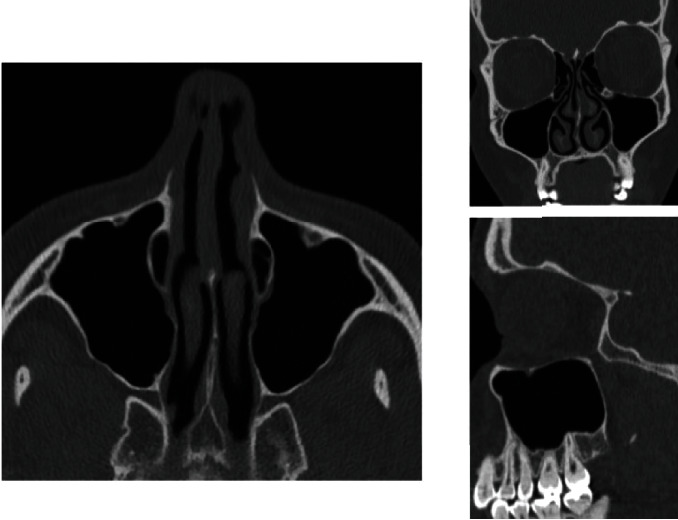
CT scan of the maxillary sinuses showing the anatomy of the sinuses and their relationship to the teeth.

Oroantral communication (OAC) is a gap between the maxillary sinus and the oral cavity. When left untreated, it can lead to an oroantral fistula and maxillary sinusitis [1]. Oroantral communications are often iatrogenic following the extraction of antral or sinus teeth. Indeed, the inferior wall of the maxillary sinus has an anatomical relationship with the maxillary premolar area (antral teeth) (Figure 1). The distance between the apex and the sinus is 1 to 7 mm for a sinus floor thickness of 2 to 3 mm. Thus, sinus effraction can be observed in 3.8% to 1.3% of cases after maxillary molar extraction [2]. OACs can also be induced by tumor surgery, implant surgery, trauma, or orthognathic surgery involving the maxilla.

**Figure 2:**
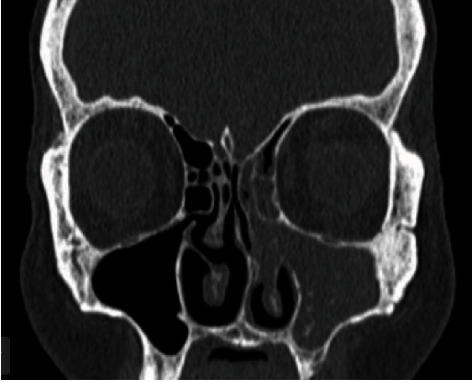
Frontal CT section showing the filling of the left maxillary sinus.

Surgical treatment of OACs should be performed as early as possible. Indeed, in the face of extensive untreated oroantral communication, 50% of patients develop sinusitis after 48 hours and 90% after two weeks with a filling of the sinus on radiological examination [3] (Figure 2). In the healthy sinus, OACs less than 5 mm close spontaneously [4]. Nevertheless, it is difficult, if not impossible, to evaluate the diameter of an OAC in the clinical setting, which is why it is necessary to intervene surgically in the majority of clinical situations. Different surgical techniques have been described, such as surrounding soft tissues (vestibular flap, palatal flap, buccal fat pad).

The high recurrence rate for significant bone defects and the increasing need to obtain closure for implant rehabilitation has led practitioners to develop new bone closure techniques using new materials that allow for new therapeutic perspectives.

The choice of the appropriate treatment depends on several factors, including the time of consultation, the presence of associated sinusitis, or the size of the communication.

This work is aimed at describing the different therapeutic options of OACs through some clinical cases and at presenting the decision factors of these treatments.

## 2. Observations

### 2.1. Observation 1

**Figure 3:**
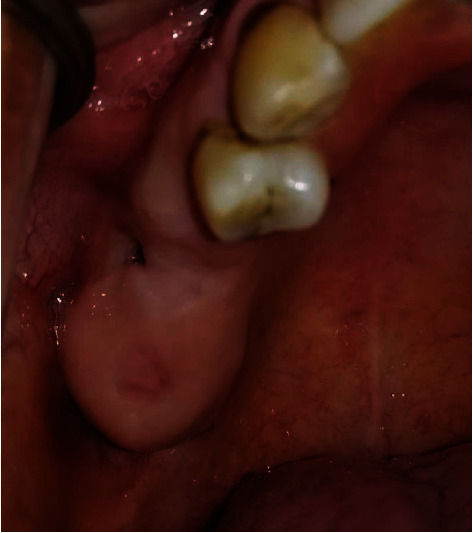
Intraoral view showing a 3 mm diameter oroantral fistula on the right maxillary ridge.

**Figure 4:**
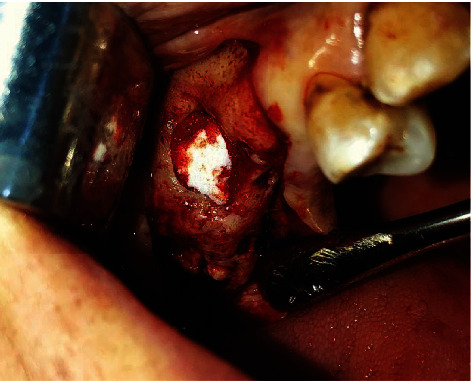
Intraoral view showing the closure of the OAC by a collagen membrane.

A 41-year-old female patient with no particular medical history was referred to the Surgical Odontology Department of the Casablanca Dental Consultation and Treatment Center by her private dentist following the persistence of a right maxillary oroantral fistula (OAF) for one month. The patient reported pain in the right upper maxillary area accentuated by gravity and associated with oronasal fluid reflux. The extraoral examination was unremarkable. The intraoral examination showed a fistula about 3 mm in diameter on the right posterior maxillary ridge with normal mucosa (Figure 3). The panoramic radiograph showed a bony discontinuity in the fistula area with a relationship to the right maxillary sinus. We confirmed the diagnosis of oroantral fistula associated with maxillary sinusitis. The management consisted initially in the sanitation of the maxillary sinus, which an otolaryngologist performed. The surgical treatment for closure of the OAC consisted of traction of a vestibular flap following the Rehrmann technique associated with a resorbable collagen membrane (Figure 4).

**Figure 5:**
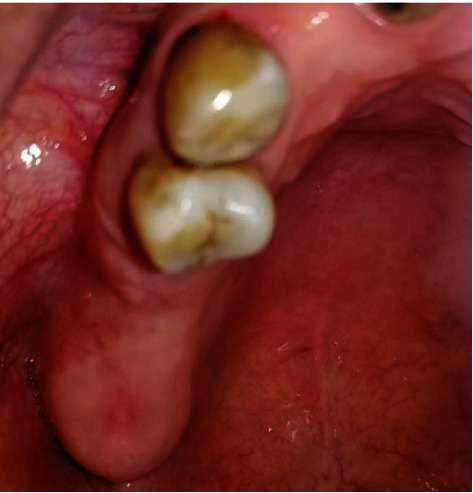
Intraoral view showing healing at one month.

Follow-up at 15 days and then at one month showed good mucosal healing and disappearance of clinical signs (Figure 5). At six-month follow-up, the patient did not present any clinical symptoms.

### 2.2. Observation 2

A 35-year-old patient presented to the Oral Surgery, Clinical Department of the Consultation Center and Dental Treatment in Casablanca, following complications of extraction of the upper left second molar, performed in a liberal dental office. The patient reports air passage via the extraction site to the nasal cavity associated with slight pain in the upper left infraorbital region accentuated by gravity. The extraoral examination was without particularity. The intraoral examination showed a persistent fistula of about one centimeter opposite the vestibular site's extraction site. The panoramic X-ray showed a bone discontinuity in the left posterior maxillary zone, which is intimately related to the left maxillary sinus. The medical care consisted initially in the sanitation of the maxillary sinus by medical treatment based on amoxicillin/clavulanic acid at a rate of 3 g per day for ten days. We managed this case using a Rehrmann flap associated with the buccal fat pad graft. Follow-up of the patient at six months and one year reported clinical silence.

### 2.3. Observation 3

A 53-year-old patient presented to the Oral Surgery, Clinical Department of the Consultation Center and Dental Treatment in Casablanca, following complications from the extraction of a maxillary wisdom tooth from a week ago, performed in a liberal dental office.

The patient reports a passage of air and food between the oral and nasal cavity, associated with pain in the left infraorbital area accentuated by gravity. In addition, the patient was treated with antibiotics at the rate of 2 g of amoxicillin a day for four days. The extraoral examination was in an unremarkable condition.

**Figure 6:**
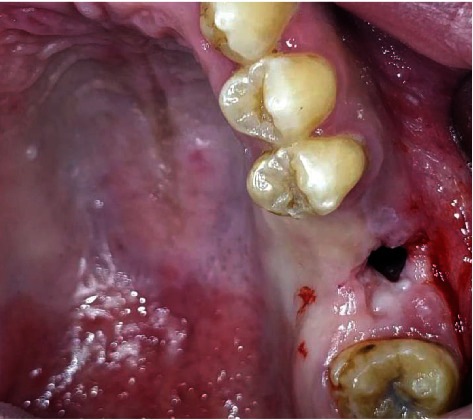
Intraoral view showing a left maxillary OAC of 7 mm diameter.

**Figure 7:**
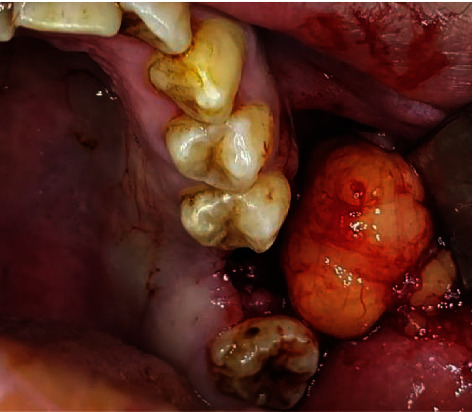
Intraoral view showing access to the buccal fat pad in treating a left maxillary OAC.

The intraoral examination demonstrated that the socket of the left maxillary wisdom tooth was persistent and with serosity all around (Figure 6). The panoramic X-ray has shown a residual root-like radioopacity of the left maxillary quadrant and a discontinuity of the bone cortex at this level, responsible for a close relationship between the oral cavity and the left maxillary sinus. The treatment consisted initially of medical treatment associated with daily nasal wash and rinse of OAC. After the signs of infection had disappeared, the surgical closure of the OAC, as in the previous case, consisted of performing a Rehrmann flap associated with a buccal fat pad graft (Figure 7). After one month and then six months, there was a complete cure and no clinical symptoms.

### 2.4. Observation 4

**Figure 8:**
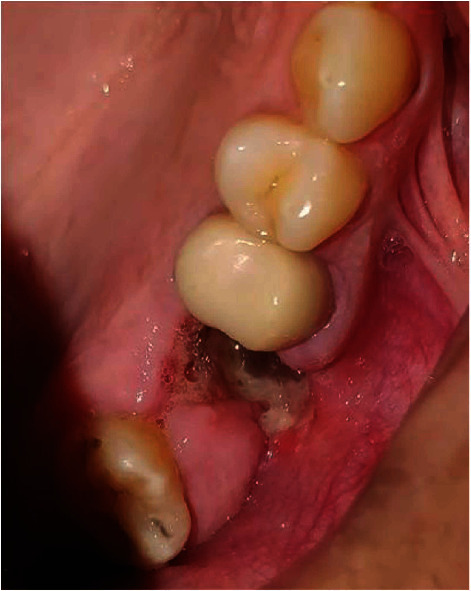
Intraoral view of a recent purulent left maxillary OAC.

A 34-year-old patient presented in consultation to the Oral Surgery, Clinical Department of the Consultation Center and Dental Treatment in Casablanca, three days after the extraction by her dentist practicing in the liberal sector. The patient reports having had a first attempt to extract her first left maxillary molar a week earlier, with persistent roots. During the interrogation, we noted that the oroantral communication (OAC) appeared during a second intervention by her doctor for extraction of the residual roots four days later. The patient reported the passage of air through the dental socket during respiratory exhalation and a slight feeling of heaviness concerning the left sinus. The extraoral examination has not shown anything unusual. The intraoral view showed a fistula of 10 mm with exposed bone (Figure 8). The Valsalva maneuver highlights the presence of bubbles in the bottom of the tooth socket. The radiological examination shows continuity between the dental socket and the left maxillary sinus.

The patient had received medical treatment based on amoxicillin at a rate of 3 g per day and 1.5 g of metronidazole per day.

**Figure 9:**
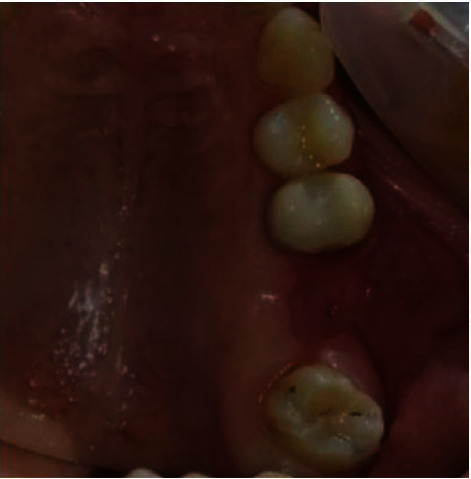
Intraoral view showing healing at 40 days.

On the other hand, the patient's fear of undergoing surgery, given her history, and the delay in consultation, led us to opt for a nonsurgical, conservative technic. After a thorough site cleaning, the fistula was closed by a eugenol-free periodontal dressing type COE-PAK∗. The patient came back regularly to change the dressing until the area healed entirely and there were no clinical symptoms after 40 days (Figure 9). After one year of follow-up, the patient did not present any clinical signs.

## 3. Discussion

Several techniques can treat OAC. the choice of method must take different factors into account: the size of the communication, the presence or absence of an infection, and, especially, the time of exposure.

Indeed, in the presence of a healthy sinus, when the treatment is early (within 48 hours), the management will only be surgical to close the OAC. In the case of deferred care, treatment has two stages:

First of all, medical treatment to cleanse the sinus [5]. Some authors recommend the following protocol: antibiotic therapy: association of amoxicillin/clavulanic acid twice a day; clindamycin 300 mg 4 times a day or moxifloxacin 400 mg once a day for at least ten days combined with a decongestant and rinse of the sinus with physiological serum through OAC [3]. Other treatment methods are possible and must be discussed in collaboration with an otolaryngologist. We will not develop this aspect of care in this work.

On the other hand, with the cleaned sinus, the treatment is surgical, closing the OAC. There is no consensus regarding a specific technique. Each of these techniques has its pros and cons. It is therefore essential to choose the method according to the clinical situation.

Before describing the different surgical options, it is essential to remember that in the presence of a healthy sinus, OACs smaller than 5 mm tend to close spontaneously [4]. Therefore, after tooth extraction, when an OAC is suspected, it would be wise to suture the surgical site with or without collagen sponges. The patient is advised to avoid all iatrogenic movements, such as the violent blowing of the nose, which could increase the size of the OAC.

### 3.1. Rehrmann's Flap

The coronally advanced flap is the most common and oldest technique known in the treatment of OAC [3, 6]. It was described by Rehrmann in 1936 and remains today the most accepted technique by the authors [7]. This technique consists of making a vestibular flap of trapezoidal shape advanced coronally to close the bone defect. Its broad base would provide the necessary vascular supply for the success of this technique. This technique has a success rate of 93% [7]. The disadvantages of this technique are postoperative pain and edema associated with a reduction in the depth of the vestibule concerned. Indeed, a second vestibule deepening surgery is often necessary after six months unless you opt for an implant solution.

### 3.2. Buccal Fat Pad Graft

This technique is suitable for small and medium volume OAC. The Buccal fat pad graft is located between the masticatory muscles with three arterial trunks to ensure vascularization: the superficial temporal, maxillary, and facial arteries. One of its extensions, called the buccal extension, is close to the maxillary premolar-molar zone. The size of this ball is constant regardless of the body mass [7]. This technique was described for the first time by Egyedi in 1977 [8]. After making a full-thickness vestibular flap around the OAC, it consists of incising in the periosteum in the posterior zone opposite the maxillary tuberosity to discover the buccal extension of the Bichat ball. This ball of fat is pulled and sutured to cover the OAC [3]. The produced flap is sutured and repositioned in its starting position. This ball of pedunculated fat left bare in the oral cavity has been shown to epithelialize after two weeks [9]. This technique has the advantage of keeping the vestibule intact. However, the buccal fat pad may necrose. Egyedi had recommended for this purpose to cover the buccal fat with a free skin graft [3, 8]. Other variants of this technique are informed, in particular the double thickness closure technique. It consists of covering the buccal fat of the cheek with the buccal mucoperiosteal flap [10].

We have presented this technique in two of the patients presented in this work, and it reports promising results. Nevertheless, it is essential to note that the amount of fat tissue obtained can be variable. According to Visscher et al. [7], this quantity is constant regardless of the patient's body weight, so that this variability may depend on the operator's clinical experience. For this reason, we recommend reinforcing the buccal fat pad graft with a Rehrmann flap whenever possible. This method also has the advantage of limiting the possible necrosis associated with the exposure of the buccal fat pad to the oral cavity.

### 3.3. Autogenic Bone Graft

In 1969, Proctor. was the first to suggest the use of autogenous iliac bone grafts in the closure of large OACs [7]. Given the additional costs and comorbidity linked to this technique, it was abandoned. Haas et al. recommend using monoblock bone grafts for the closure of OAC [11]. These blocks are prepared to adapt to the bone defect and remain stable. Otherwise, they will have to be stabilized using miniscrews.

A Rehrmann-type vestibular flap covers both the bone defect and the graft.

### 3.4. Allogenic and Xenogenic Materials

Some authors use these materials for closing oral communications. Marković reported a case of OAC treated with a collagen membrane. After completing a mucoperiosteal vestibular flap and elimination of the mucosal pathway of the fistula, the technique consists of placing the membrane on the bone defect. The flap is thus returned to its original position without covering the collagen membrane [12]. This technique differs slightly from the first case reported in this work, where the mucoperiosteal flap covers the membrane. We placed an allogenic membrane that we covered with the vestibular flap drawn coronally. Clinical experience has shown us an increased risk of secondary infection when the membrane is exposed to the oral cavity, hence the decision to cover it entirely with the mucoperiosteal flap. This method is known as a double-layer closure technique. It is close to the triple-layer closure technique reported by George [13], which closes an oroantral communication by three levels of structure: the mucoperiosteal flap associated with a buccal fat pad and a fibrin membrane rich in platelets. The cost of this therapy remains a critical limit.

On the other hand, OAC may be effectively closed using lyophilized fibrin glue of human origin. After preparing fibrin for 15 to 20 minutes, it is placed at the level of the OAC associated with a collagen membrane [7]. The advantage of this technique is the absence of a flap and, therefore, of postoperational effects. In the same way, the anatomy of the oral cavity also remains intact. On the other hand, the risk of disease transmission and the preparation time are significant drawbacks.

### 3.5. Laser Therapy

The use of laser by the LLLT (low-level laser therapy) technique has also been reported in the treatment of OAC. Grzesiak-Janas and Janas reported using a laser with a wavelength of 830 nm with power at 30 mW in continuous mode at the rate of 3 cycles of intraoral and extraoral. The same cycle is repeated for four consecutive days [14].

The cost and lack of clinical hindsight of this technique come as a significant limit.

### 3.6. Conservative Nonsurgical Treatment

In certain circumstances, surgical treatment turns out to be difficult or even impossible for the closure of OAC. In patients with general conditions or pathologies leading to immunosuppression, postoperative infection is more significant, and scarring may be altered. Logan and Coates reported a case of conservative treatment in a patient presenting with an HIV infection [15]. The authors used a surgical splint such as a palatal plate made of acrylic resin covering the defect. After continuously wearing the plate for two weeks, satisfactory results were visible. After eight weeks, the authors emphasize the complete healing of the bone defect. This technique is similar to the one used in the last case reported in this work. Indeed, the difference is using an alloplastic material (COE-PACK) to isolate the defect instead of a palatal plate locally. This conservative technique has significant advantages but requires good patient cooperation, given the healing time and the discomfort.

Faced with this plethora of techniques, it can be challenging to choose a good strategy. For this reason, we propose some recommendations based on the clinical situation (Table 1).

## 4. Conclusion

Today, there is no best treatment or consensus regarding oroantral communication. Each technique has its advantages and disadvantages. The choice of a method must be made on a case-by-case basis, considering all factors regarding the act and the patient. According to Visscher et al. [7], the ideal treatment for OAC is fast, secure, simple, and well-tolerated by patients, at low cost, resulting in good bone and mucosal healing.

## Figures and Tables

**Table 1:** Recommendations for the choice of surgical techniques according to the clinical situation.

Clinical situation			Surgical technique
Suspicion of OAC postextraction			Collagen sponges + sutures

Confirmed OAC	Unplanned implant^∗^	<10 mm of diameter	Rehrmann's flap or conservative nonsurgical technique
		10-15 mm of diameter or failure of anterior treatment	Buccal fat pad
		>15 mm of diameter or failure of anterior treatment	Combination of techniques: double or triple thickness closure technique: (i) Buccal fat pad + Rehrmann's flap (ii) Buccal fat pad + allogenic membrane + Rehrmann's flap
	Planned implant^∗^	Autogenic or xenogenic bone graft	

^∗^The implant placements described in the table are for the exact areas of the OACs without considering the probably healthy adjacent areas.
